# Traits across trophic levels interact to influence parasitoid establishment in biological control releases

**DOI:** 10.1002/ece3.8654

**Published:** 2022-03-08

**Authors:** Benjamin J. M. Jarrett, Marianna Szűcs

**Affiliations:** ^1^ 3078 Department of Entomology Michigan State University East Lansing Michigan USA; ^2^ 3078 Department of Biology Lund University Lund Sweden

**Keywords:** biological control, generalist, herbivore, host range, invasion biology, specialist

## Abstract

A central goal in ecology is to predict what governs a species’ ability to establish in a new environment. One mechanism driving establishment success is individual species’ traits, but the role of trait combinations among interacting species across different trophic levels is less clear. Deliberate or accidental species additions to existing communities provide opportunities to study larger scale patterns of establishment success. Biological control introductions are especially valuable because they contain data on both the successfully established and unestablished species. Here, we used a recent dataset of importation biological control introductions to explore how life‐history traits of 132 parasitoid species and their herbivorous hosts interact to affect parasitoid establishment. We find that of five parasitoid and herbivore traits investigated, one parasitoid trait—host range—weakly predicts parasitoid establishment; parasitoids with higher levels of phylogenetic specialization have higher establishment success, though the effect is marginal. In addition, parasitoids are more likely to establish when their herbivore host has had a shorter residence time. Interestingly, we do not corroborate earlier findings that gregarious parasitoids and endoparasitoids are more likely to establish. Most importantly, we find that life‐history traits of the parasitoid species and their hosts can interact to influence establishment. Specifically, parasitoids with broader host ranges are more likely to establish when the herbivore they have been released to control is also more of a generalist. These results provide insight into how multiple species’ traits and their interactions, both within and across trophic levels, can influence establishment of species of higher trophic levels.

## Graphical abstract

Understanding how communities assemble is a major question in ecology. There has been little focus on how species interactions across trophic levels may influence the establishment of a species in a new community. We analyzed a dataset of deliberate introductions of parasitoids as biological control agents to fill this gap. We found that traits of the parasitoid species and their herbivorous hosts interacted to influence the establishment success of the parasitoid. Generalist parasitoid species were more likely to establish in a community when their hosts were also generalists. On the other hand, specialist parasitoids were equally likely to establish on specialist or generalist herbivores.

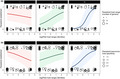

## 1 INTRODUCTION

Biological invasions have provided great insights into the numerous ecological, evolutionary, and environmental factors that can influence establishment success of different species in new regions (Dlugosch & Parker, 2008; Kolar & Lodge, 2001; Lockwood et al., 2005; Maintner et al., 2012; Sherpa & Després, 2021). In turn, these insights have contributed to a better understanding of community assembly, rapid evolution, and range expansion of species (HilleRisLambers et al., 2012; Lee, 2002; Phillips & Perkins, 2019; Sax et al., 2002, 2007; Seebens et al., 2017; Sherpa & Després, 2021). Species characteristics are one of the factors that can play a role in establishment (Hayes & Barry, 2008; Kolar & Lodge, 2001). For example, species of pine (*Pinus*) with smaller seeds, a shorter juvenile period, and shorter interval between large seed crops are more likely to invade a community (Rejmánek & Richardson, 1996), and invasive fish species are more likely to be large species that mature late, with few spawnings per year (Vila‐Gispert et al., 2005), but few general patterns of traits linked with invasion success were found to hold across taxa (Hayes & Barry, 2008).

In a new environment, invading species interact with the other species in the trophic levels above and below them, as they fit into a new ecological network. It is less well understood how specific traits interact with traits of species in a different trophic level to influence the likelihood of a successful invasion (but see Bailey et al., 2009, Paynter et al., 2012 on how traits across trophic levels might structure communities). Commonly, phylogenies of species occupying the same trophic level have been used to assess trait similarity, as species that are more closely related are assumed to have more similar trait values than species that are more distantly related (Tucker et al., 2018). Using the networks across four trophic levels, Elias et al. (2013) found that phylogeny of the host was crucial in determining what species consumed it, but at higher trophic levels, closely related consumer species did not utilize the same hosts. These network links, in addition to within‐trophic level competition, determine the outcomes of the invasion and therefore whether a newly introduced species establishes or not. Interactions across trophic levels are also crucial in determining establishment of higher trophic levels in cynipid gallwasp and parasitoid systems on oak trees (Bailey et al., 2009). Cynipid species that induce galls with similar toughness, stickiness, and hairiness support similar parasitoid communities that are adapted to attack particular types of galls, an effect that influences generalist parasitoids to a greater degree than specialists (Bailey et al., 2009). Invasion of a new gallwasp parasitoid into such a community would therefore depend on the traits of the gallwasps already within the community and the characteristics of galls they create. Such dynamics are important to understand and to predict, both for conservation and applied practices, as parasitoids invading a new community can quickly dominate a food web (Henneman & Memmott, 2001).

One way to test how biotic interactions that span trophic levels influence the likelihood of a successful invasion is by studying a special case of biological invasion: importation biological control (Hawkins et al., 1999). Importation or classical biological control entails deliberately releasing natural enemies into a community to control an invasive pest organism and can be considered “deliberate community assembly” (Holt & Hochberg, 2001). The majority of introduced biological control agents have a history of coevolution with the pest they are released to control and are primarily chosen based on diet breadth and for their potential to provide pest control. This is not the case for biological invasions more generally, where preselection based on trait values might be limited to dispersal and chance. Using importation biological control to study establishment success, however, overcomes two problems of studying biological invasions (Mills, 2001; Rossinelli & Bacher, 2014; Stiling, 1990). First, invasions lack a predicted outcome and mechanism, due to the paucity of information about how an invasive species might fit into an ecological network. In importation biological control, the biological control agent is released to combat a specific pest, providing a prediction for exactly what the key trophic interaction of the exotic species will be, and therefore how it will fit into the existing network. The second problem is that most of the available data concern successful invaders and attempted invasions that do not result in establishment are rarely recorded. In importation biological control, however, these introductions have measured outcomes: establishment or no establishment of the biocontrol agent, which allows more comprehensive analyses to be performed on the factors that influence invasion success (Van Driesche et al., 2018; Winston et al., 2014).

We specifically focus on the importation biological control releases of parasitoids, which are the most commonly used biocontrol agents against exotic insect herbivore pests (Greathead & Greathead, 1992; Stiling & Cornelissen, 2005). Establishment of parasitoids and other natural enemies introduced for biocontrol is at under 33% worldwide (Cock et al., 2016) and around 54% in North America (Van Driesche et al., 2020), which, while relatively successful, shows that we still have a lot to learn about the factors that may be important for establishment into a new environment. For biocontrol releases specifically, Rossinelli and Bacher (2014) showed that dietary specialization had a strong effect as specialist parasitoids were more likely to establish than generalist parasitoids. In an earlier study, establishment success of specialists and generalists did not significantly differ, though specialists tended to establish at higher rates (Stiling, 1990). There is evidence that some parasitoid traits, like whether they were gregarious or solitary, can explain variation in establishment success during biocontrol releases (Mills, 2001; Rossinelli & Bacher, 2014; Stiling, 1990). So, too, may host traits; for example, in importation biological control programs against weeds, Paynter et al. (2012) found that the traits of the invasive weed increased the likelihood of a natural enemy establishing, especially if the weed was an asexual, aquatic species. For parasitoids, the taxonomy of their target host and their fecundity, voltinism, mobility, feeding range, host range, and habitat are all suggested to influence their establishment (Stiling, 1990).

The above studies show some emerging patterns, especially regarding the differences in the ability of parasitoids that differ in their levels of specialization to establish. To better understand the process of community assembly, however, the interactions between the traits of the parasitoids and their hosts should be considered and not each on their own. Heimpel and Mills (2017) highlight that some host characteristics, such as, the stage of host that is most vulnerable, should be taken into account when choosing a natural enemy, which can be guided by theoretical models that incorporate stage‐specific processes (for example, Godfray & Waage, 1991).

Here, we address the importance of both parasitoid traits and herbivore traits in establishment success by analyzing importation biological control releases. To do so, we take advantage of the latest dataset detailing importation biological control introductions, which provides information on all insect species introduced to control other herbivorous insect pests in North America between 1985 and 2018, as well as the establishment success of each species, their host specificity, and the species they were released to control (Van Driesche et al., 2018). We supplemented this dataset by including five additional parasitoid traits (phylogenetic host range, developmental stage the parasitoid attacked, idio‐/koinobiont, endoparasitoid/ectoparasitoid, and solitary/gregarious) for 132 parasitoid species and five herbivore traits (host range, univoltinism/multivoltinism, size, number of eggs in an egg mass, and number of developmental stages or instars) for the 67 herbivore species they were released to control, as sometimes multiple parasitoid species were released to control the same pest species. We also collected information about the residence time of the herbivore prior to the release of biological control agents. We then evaluated the predictive value of individual parasitoid and herbivore traits, as well as multiple interactions between parasitoid and host traits predicted to contribute to establishment success, which are summarized in Table 1.

**TABLE 1:** Summary of the host and parasitoid traits and specific interactions among them that were investigated, and the predicted outcomes for each

Traits	Predictions	
(a) Parasitoid traits		
Host range	Parasitoid species with a larger host range will be more likely to establish (Vasquez, 2006) due to greater availability of possible hosts in time and space	
Taxonomic host specificity	Parasitoid species attacking hosts that are sparsely distributed across a phylogeny will be more likely to establish (Vasquez, 2006) due to greater availability of hosts in time and space	
Host stage attacked	Parasitoid species that can attack earlier host developmental stages (i.e., eggs or smaller sized nymphs) more likely to establish as they may be able to outcompete other parasitoids (Murdoch et al., 1996)	
Idiobiont or koinobiont	Idiobionts will be more likely to establish, as they are more likely to be generalists (Hawkins et al., 1990)	
Endoparasitoid or ectoparasitoid	Endoparasitoids will be more likely to establish as they have specific adaptations to find hosts in their early developmental stages (Harvey et al., 2013)	
Solitary or gregarious	Gregarious parasitoids will be more likely to establish since they lay multiple eggs within a patch that increases chances that at least a few individuals may develop successfully (Mills, 2001)	
(b) Herbivore traits		
Host range	Specialist invasive herbivores often attack crops and less likely to have trophic links with native plant species; therefore, parasitoids introduced on the specific target crop are likely to establish (Hawkins et al., 1990)	
Voltinism	Parasitoids are more likely to establish on hosts that can complete more than one generation a year given the availability of resources for longer period	
Size of developmental stage attacked	Parasitoid species can display size‐dependent parasitism rates (Murdoch et al., 1996), and thus, larger herbivores may increase the probability of parasitoid establishment	
Number of eggs in an egg mass	Herbivores with larger numbers of eggs within an egg mass will increase the probability of parasitoid establishment because of larger patch size and increased host density (e.g., Hassell, 1982)	
Number of developmental stages	The larger the number of instars of the host, the more likely a parasitoid can establish due to longer availability of hosts for parasitism	
Residence time	Negative relationship of parasitoid establishment success with host residence time due to accumulation of natural enemies (competitors) and local adaptation of the host	
(c) Interaction of parasitoid and herbivore traits		
*Parasitoid traits*	*Herbivore traits*	
(Taxonomic) host range	Host range	We predicted that specialized parasitoids are more likely to establish when their host is also a specialist as parasitoids may be also coevolving with signals from the hosts of the target herbivore species (Abdala‐Roberts et al., 2019; Price et al., 1980; Turlings & Erb, 2018; Vet & Dicke, 1992). On the other hand, generalist parasitoids are expected to establish independently of herbivore host range, given the wide breadth of hosts they may be able to utilize in various environments (Symondson et al., 2002)
Solitary/gregarious	Size	We predicted that gregarious parasitoids would be more likely to establish on larger hosts because those would provide more resources for the multiple offspring they produce per host, as some gregarious species show the capacity to alter their clutch size in response to larger hosts (Bezemer & Mills, 2003)
Idio/koinobiont	Host range	We predicted koinobionts are more likely to establish on generalist herbivores (Kirichenko et al., 2013) and idiobionts are more likely to establish on specialist herbivores
Endo/ectoparasitoids	Host range	External feeders are more likely to be generalists (Kirichenko et al., 2013) and so offer opportunities for parasitism to ectoparasitoids that may not be afforded to endoparasitoids (Gauld, 1988). We therefore predict ectoparasitoids are more likely to establish on generalist herbivores
(Taxonomic) host range	Residence time	We predicted that generalists would have greater competition from native generalist natural enemies that tend to accumulate on invasive hosts when the host has been in their introduced range for a longer period (Broadley et al., 2018; Cornell & Hawkins, 1993)

## 2 METHODS

We extracted information on establishment success and host range of parasitoids from a recently updated database on biological control releases against insects in North America from 1985 to 2018 (Van Driesche et al., 2018). In Van Driesche et al. (2018), parasitoids from the Aphelinidae (number of species = 27), Bethylidae (*N* = 2), Braconidae (*N* = 30), Chalcididae (*N* = 1), Encyrtidae (*N* = 21), Eulophidae (*N* = 17), Ichneumonidae (*N* = 8), Mymaridae (*N* = 3), Platygastridae (*N* = 5), Proctotrupidae (*N* = 1), Pteromalidae (*N* = 4), Scelionidae (*N* = 1), Tachinidae (*N* = 5), and Trichogrammatidae (*N* = 7) were included. We then searched the literature for life‐history traits for each parasitoid species and their herbivore targets (see Supplementary Data for references). For parasitoids, we included four additional traits besides host range that was indicated in the database (all of which are defined in the Methods below): the host stage attacked; whether the species was an idiobiont or a koinobiont; an ecto‐ or endoparasitoid; and solitary or gregarious. For the hosts, we added five traits: host range, voltinism, size of developmental stages, numbers of eggs in an egg mass, and the number of developmental stages.

We focused on the four major orders of herbivorous pests: Hemiptera (*N* = 84 releases), Lepidoptera (*N* = 37 releases), Coleoptera (*N* = 29 releases), and Diptera (*N* = 11 releases). We excluded two orders (Orthoptera and Thysanoptera) because there were too few releases targeting them (three and two respectively). There were 11 releases against Hymenopteran pests, but these included eusocial ant species that are not herbivorous (and so do not have the same trophic links as herbivorous pests) and were thus excluded.

In this paper, we analyzed parasitoid establishment and not the primary outcome of biocontrol releases, pest suppression, and control. Establishment success, the response variable for our analyses, was determined by Van Driesche et al. (2018) by assessing the literature or through personal communication with experts involved in the release of the biological control agent and its subsequent monitoring. If the biological control agent was found in traps the years following the release, the agent was deemed to have established. This metric does not take into account variation in establishment (e.g., population size over time and time to establishment) and so creating a binary variable from a process that is most likely not binary may have problems and not capture the true variation in establishment success in nature. This is a form of vote‐counting (Haddaway et al., 2020) and can result in potential erroneous 0s through low statistical power or insufficient trapping and thus may be underestimating any effects we find in our analysis. While this is far from optimal, monitoring techniques vary depending on the insect species in question, and condensing such variation in the type of data generated may currently be the best way to draw broad conclusions from biological control releases. For more rigorous tests examining invasion success, we urge practitioners to standardize efforts for assessing whether a biological control agent has established or not.

We chose to focus on establishment and not control for two reasons: first, quantifying control of a herbivore pest is more difficult to assess and standardize across parasitoids and pest species than establishment; and second, if a parasitoid established in our dataset, it often led to some level of control as establishment and effectiveness can be highly correlated (Rossinelli & Bacher, 2014; Stiling, 1990). In this dataset, 71 parasitoids established (at least once) and 61 parasitoid species failed to establish. Of the 71 established parasitoids, Van Driesche et al. (2018) counted 38 as successfully controlling the pest they were released to control, nine have failed to control the pest, with uncertainty of the impact of the remaining 24 species.

### 2.1 Parasitoid traits

#### 2.1.1 Host range

For the host range of the parasitoids (the number of species, genera, and families on which they are known to successfully reproduce), we used the data from Van Driesche et al. (2018) that details the known host range of the parasitoid agents (number of species used in this analysis = 98). The sample size is indicated in parentheses at the end of the opening sentence describing each trait or interaction. We extracted the number of families, the number of genera, and the number of species the parasitoid is known to attack. These measures are significantly correlated: the number of families and genera attacked (Pearson's correlation: *r*_138_ = .52, *p* < .001); the number of families and species attacked (*r*_137_ = .40, *p* < .001); and the number of genera and species attacked (*r*_137_ = .92, *p* < .001). Instead of focusing on the number of species a parasitoid can attack, we used the number of genera a parasitoid can attack as our numerical estimate of taxonomic host range. The number of genera a parasitoid can parasitize is more robust to new records of parasitism and so suffers less when our knowledge of host range is imperfect; additional records of parasitism will always change host range estimates constructed from the number of species or any phylogenetically or taxonomically informed metrics. Numerical metrics, however, will always inflate the host range of a species as it treats each genus independently, which is why having multiple host range metrics is a pragmatic approach for such analyses. The parasitoid species used in this study attacked an average of 1.59 families (range: 1–14), 4.60 genera (1–31), and 7.80 species (1–71), with histograms of host range shown in the Appendix S1 (Figure S1).

#### 2.1.2 Phylogenetic host range

The number of genera a parasitoid can attack as a host range metric is problematic, as it does not take phylogeny into account (Abram et al., 2021; Heimpel et al., 2021). A parasitoid that parasitizes three genera in the same subfamily is different from one that parasitizes three genera in three different families, for example. We therefore also calculated the taxonomic host specificity index advocated by Poulin and Mouillot (2003) (*N* = 95). This index is computed as follows:

$$S_{\mathrm{DT}}=2\frac{\sum \sum_{i<j}\omega_{\mathrm{ij}}}{s\left(s‐1\right)}$$

where s is the number of host species a parasitoid can attack, and ω_ij_ is the taxonomic distance between a pair of host species (which is equal to 1 if they are in the same genus, 2 in the same family, and 3 in the same order). This metric takes the number of species a parasite or parasitoid can attack and how related these species are and is bounded by 1 where all host species are in the same genus, and 3 where all host species are in different orders. This bounding is not something that applies to numerical estimates of host range, where the number of genera can increase almost without limit, and so again may overinflate host range estimates. S_DT_ is a metric that also performs well relative to other taxonomically informed metrics of host range (Abram et al., 2021) and complements numerical estimates of host range. While we expect the number of genera and S_DT_ to align, differences in the results obtained by these two metrics might arise. If S_DT_ does not show any patterns but the numerical host range based on the number of genera attacked does, we would conclude that taxonomic‐based nonindependence in host range constrains establishment probability. If S_DT_ is important in predicting establishment success, but the number of genera is not, we might conclude how the host range of a parasitoid is structured taxonomically is far more influential in governing establishment success. The parasitoids used in this study had an average phylogenetic host range of 1.64 (range: 1–3).

#### 2.1.3 Host stage attacked

We used four categories to define this trait: egg (number of egg parasitoids in the dataset, *N* = 20), instar (nymph or larva, *N* = 67), pupa (*N* = 4), and adult (*N* = 5, total *N* = 96). Instar parasitoids attack the juvenile stages of any insect, which includes the larval stages of holometabolous insects and the nymphal stages of hemimetabolous insects. Parasitoids were classified as pupal parasitoids if they also parasitize prepupae. If a parasitoid species was recorded as parasitizing more than one developmental stage, we recorded it as a parasitoid of the earliest developmental stage. Parasitoids that can attack earlier developmental stages (i.e., eggs or smaller sized nymphs) may be more likely to outcompete other parasitoids and be more likely to invade a community (Murdoch et al., 1996).

#### 2.1.4 Idiobiont or koinobiont

An important life‐history strategy used to classify parasitoids is whether they are an idiobiont or a koinobiont (Godfray, 1994) (*N* = 91). Idiobiont parasitoids paralyze their hosts, so the hosts cease developing, while koinobiont parasitoids lay their eggs in hosts, which continue to develop only to be consumed by the parasitoid larva later in development. We predicted that idiobionts would be more likely to establish as they are more likely to be generalists (Hawkins et al., 1990). However, the species contained in our dataset do not show this idiobiont‐generalist correlation (see Appendix S1, Figure S2).

#### 2.1.5 Endoparasitoid or ectoparasitoid

Endoparasitoids lay their eggs inside their host while ectoparasitoids lay their eggs outside the host (Godfray, 1994) (*N* = 104). Larvae of ectoparasitoids may burrow into the host or continue to feed outside the host, and they are more likely to be idiobionts. Endoparasitoids are thought to be ecologically superior to ectoparastioids, as endoparasitoids are more specialized in finding numerous early‐development hosts (Harvey et al., 2013).

#### 2.1.6 Solitary or gregarious

We classified parasitoid species as solitary when they lay a single egg into a host and gregarious when the parasitoid lays more than one egg into a single host (Godfray, 1994) (*N* = 97). This means we included parasitoid species that laid more than one egg into a host but only one larva emerges as gregarious. Mills (2001) found that gregarious parasitoids were more likely to result in successful biological control introductions.

The above parasitoid traits on which we have focused are not independent (Mayhew & Blackburn, 1999). Idiobionts tend to be ectoparasitoids that are generalists, whereas koinobionts tend to be endoparasitoids that are specialists (Quicke, 1997). In our dataset, we have found this largely to be true (Appendix S1, Figure S2), though the correlation is not perfect; therefore, there is added value to including these variables separately. The collinearity of parasitoid traits can inflate regression estimates of paired variables that are correlated, and thus, we only used one parasitoid trait in each analysis.

### 2.2 Herbivore traits

#### 2.2.1 Host range

Similar for parasitoids, we quantified the target herbivore's host range (*N* = 111). Ideally, we would have used the same metrics of host range on the herbivore data than we did for estimating parasitoid host range. Unfortunately, data on the number of species of plants a herbivore feeds on are usually incomplete, and so, we only included the number of families on which the pest can feed, which are data that are readily available for the wide range of insect herbivores included in our dataset. Hawkins et al. (1990) found that top‐down control of invasive herbivorous pests is more likely in simplified trophic systems involving exotic crop species. Specialist herbivore species are therefore less likely to have trophic links with native plant species and so might be expected to have their biological control agent establish (Hawkins et al., 1990).

#### 2.2.2 Voltinism

Voltinism refers to the number of generations a species can complete in one year (*N* = 104). In many insect species, it is a plastic, temperature‐dependent trait. To reduce clinal variation due to temperature, we classified herbivore species as either obligately univoltine (they can only complete a single generation a year) or multivoltine (where they show the capacity in some areas of their range to complete more than one generation a year).

#### 2.2.3 Size of developmental stages

The size of a host is an important determinant of parasite fitness, as larger hosts yield larger, or more, offspring (Waage, 1986). Parasitoid species may also display size‐dependent parasitism rates that may alter the likelihood of establishment (Murdoch et al., 1996). We included adult, pupal, and instar sizes as length measurements when they could be found (*N* = 67). We included only the size of the last instar, as it is the stage with the greatest variation in size, and this information is most commonly available. We used a database on egg sizes for data of most herbivore species in our database (Church et al., 2019) and conducted additional literature searches to find missing data.

#### 2.2.4 Number of eggs in an egg mass

Herbivorous insects may lay eggs singly or in a group. We found estimates in the literature that would often include a range of eggs found in an egg mass. We therefore included the minimum and the maximum number of egg masses reported. For singly laid eggs, both numbers would be one. We excluded pupal and adult parasitoids for this analysis, as this metric also likely correlated with instar density, especially in early instars (*N* = 58). Numbers of eggs in an egg mass is directly linked to the resource patch size in many papers modeling parasitoid population dynamics (e.g., Hassell, 1982), where increased host density within a patch, like increased egg numbers, results in greater levels of parasitism.

#### 2.2.5 Number of developmental stages

We collected data on the number of instars species go through, which may be regarded as an additional measure of developmental time and therefore a measure of the time of opportunity that instar parasitoids have to parasitize hosts (*N* = 71). We predicted that the larger the number of instars of the host, the more likely a parasitoid can establish. Nutrition and temperature can influence the number of molts, and thus, in cases when we found a range, we used the smallest number of molts, as that would be the minimum number of molts required to complete development.

#### 2.2.6 Residence time

In addition to herbivore life‐history traits, we also collected data on when the invasive pest species was first recorded in the geographic region the biological control agent was released (*N* = 89). We predicted that the longer the residence time of the pest, the less likely that a biological control agent will establish as the host has had more time to accumulate natural enemies that might otherwise compete with the importation biological control agent.

### 2.3 Interaction of parasitoid and herbivore traits

We tested five a priori hypotheses from the accumulated dataset, as an exploratory analytic approach is prone to p‐hacking because five parasitoid traits and five herbivore traits in combination yield a large array of possible models.

1. Does the host range of the herbivore species interact with the host range of the parasitoid species to determine establishment success? We predicted that specialized parasitoids are more likely to establish when their host is also a specialist as parasitoids may be also coevolving with signals from the hosts of the target herbivore species (Abdala‐Roberts et al., 2019; Price et al., 1980; Turlings & Erb, 2018; Vet & Dicke, 1992). On the other hand, generalist parasitoids are expected to establish independently of herbivore host range, given the wide breadth of hosts they may be able to utilize in various environments (Symondson et al., 2002). Hawkins et al. (1999) found that success rates of importation biological control occurred in simplified trophic systems, a pattern that predicts that establishment would occur when herbivores are specialists and are found in simplified agroecosystems, especially when the parasitoid released is a specialist. We asked this question for both the number of genera attacked by a parasitoid species as well as their metric of phylogenetic host range (number of genera: *N* = 95; phylogenetic host range, *N* = 92).
2. Do solitary or gregarious life histories interact with host size to influence establishment rate in the case of instar parasitoids? There is some evidence that gregarious parasitoids are more likely to establish in biological control releases (Mills, 2001) and also show some capacity to adaptively alter their clutch size in response to larger hosts (Bezemer & Mills, 2003). We therefore predicted that gregarious parasitoids would be more likely to establish on larger hosts because those would provide more resources for the multiple offspring they produce per host (*N* = 43).
3. Are koinobiont parasitoids more likely to establish on generalist herbivores? Idiobionts are frequently equated with generalists and koinobionts with specialists (Godfray, 1994; Hawkins et al., 1990), though Traynor (2004), as in our dataset, does not find such a pattern in a broad comparative analysis. We therefore analyzed whether a parasitoid was an idiobiont or koinobiont and parasitoid host range separately (*N* = 88). In addition, generalist herbivores attack more species of plant and are more likely to feed externally (Kirichenko et al., 2013). External feeding by insect herbivores is a strategy that may suit koinobionts that can freely parasitize herbivore larvae, which then complete development in a concealed place and thus offer protection for the developing wasp (Gauld, 1988).
4. Using similar logic to hypothesis 4 are ectoparasitoids more likely to establish when released to control generalist herbivores? Internal feeders are more likely to be plant specialists (Kirichenko et al., 2013) and so offer fewer opportunities for parasitism to ectoparasitoids by feeding in a concealed feeding space (Gauld, 1988) (*N* = 101).
5. Are generalists less likely to establish when their herbivore hosts have had time to accumulate native natural enemies? We tested this hypothesis by interacting the parasitoid host range (number of genera attacked, *N* = 79, and phylogenetic host range, *N* = 77) with the residence time of the herbivore species. We predicted that generalists would have greater competition from native generalist natural enemies that tend to accumulate on invasive hosts when the host has been in their introduced range for a longer period (Broadley et al., 2018; Cornell & Hawkins, 1993).

### 2.4 Parasitoid and host cladograms

The phylogenies of both the parasitoid species and the herbivore species are expected to influence the outcomes of community processes (Bailey et al., 2009; Cavender‐Bares et al., 2009; Ives & Godfray, 2006). We therefore constructed cladograms for both parasitoids and herbivores to account for nonindependence of patterns in establishment though it was not the focus of this study (see Appendix S1). The resulting parasitoid and herbivore cladograms can be found as Figure S3 and were included in all models. Analyses were robust to randomization of branch lengths (Figure S4).

### 2.5 Statistical analysis

We used establishment of the parasitoid species as our response variable. For each parasitoid host species pair, we included all releases in a binomial format such that if one parasitoid was released to control one pest species four times, but only established once, the number of trials is four and the number of successful establishments is one. We therefore used a binomial error distribution in our models with a logit link function.

We constructed one model per parasitoid or host trait, and a separate model for each interaction, totalling 18 models that equate to the rows in Table 1. The following traits were coded as binary variables for the parasitoid species: idiobiont or koinobiont; endoparasitoid or ectoparasitoid; and solitary or gregarious. For the herbivore species, voltinism was also coded as a binary variable (univoltine or multivoltine). Host range data for the herbivore were scaled and log‐transformed prior to analysis to aid model fitting. Each model was therefore fit with the following structure (eqn 1) where “trait” is replaced with the trait (or interaction of traits) of interest:

(1) $$\text{successes}|\text{releases}∼\text{trait}+\left(1|\text{parasitoid}\;\text{cladogram}\right)+\left(1|\text{herbivore}\;\text{cladogram}\right)+\left(1|\text{year}\right)+\left(1|\text{location}\right)$$

where the cladograms are included in the model as covariance matrices with Grafen branch lengths. Branch length randomization does not appear to influence our results and conclusions (Figure S4).

We report estimates of the regression estimate, *β*, with 89% credible intervals (as recommended by McElreath, 2018). If the *β* value has credible intervals that do not overlap with 0 or credible intervals close to 0 but have a large effect size, there is evidence that the effect of that trait on establishment success is not negligible and we thus performed further steps to assess the importance of the trait in question. We subsequently constructed a second, simpler model. In the case of a model with one variable, like parasitoid host range, for example, we removed that trait from the model and compared the model containing parasitoid host range with the model excluding it. We then compared the simpler model with the more complex model and calculated the difference in WAIC (Watanabe‐Akaike/widely applicable information criterion), LOO (leave‐one‐out validation), and where necessary, k‐fold cross‐validation (where k = 10) between the two models. These are metrics similar to traditional AIC that estimate how well the model would predict new data compared with a different model (Burnham & Anderson, 2004; Vehtari et al., 2017; Yao et al., 2018). If the complex and simpler model perform equally well, there is little evidence to support a more complex model. In the case of an interaction between two traits, we removed the interaction to arrive to the simpler model. We also computed the model weight, which is “an estimate of the probability of that the model will make the best predictions on new data, conditional on the set of models considered” (Burnham & Anderson, 2004; McElreath, 2018). All metrics are consistent in their choices of the best model, and so we only report the WAIC differences between models and the WAIC weights.

To control for covariance in establishment success due to relatedness between parasitoid species and herbivore species, we included the cladograms of both the parasitoid and herbivore species as random terms in every model as mixed models are best suited for dealing with two separate phylogenies (Rafferty & Ives, 2013). Bayesian models such as the types we have used here are faster for a dataset of this size, and more flexible than their frequentist alternatives, and when dealing with analyses that use phylogenies, this flexibility allows for the inclusion of multiple phylogenies and a diversity of error structures (Gallinat & Pearse, 2020; Pearse et al., 2015). We also included year as a random effect to account for temporal patterns of biological control releases, and when the same parasitoid was released in multiple years, we used the year of the first introduction. Lastly, the location of the release was included as a random term. For some species, many releases were made, including some that spanned multiple states. We therefore categorized location in North America as West (California, Washington, Oregon, and British Columbia), South (Mexico, Texas, Florida, and Arizona), East (New England), Central (Montana, Michigan, and Minnesota), and other including islands and overseas territories (Hawaii, US Virgin Islands, Guam, and Puerto Rico).

To improve convergence and prevent overfitting, we specified mildly informative but conservative normal priors centered on 0 with a standard deviation of five for the regression estimate to penalize extreme values. All models were run with four chains, which were inspected visually to ascertain model performance. We used R (3.5.1) (R Development Core Team, 2018) for all data analyses (see Appendix S1 for code). Bayesian models were created in the Stan computational framework http://mc‐stan.org/ (Carpenter et al., 2017) accessed with the R package *brms* (Bürkner, 2017) with additional functions from *tidybayes* (Kay, 2019), *modelr* (Wickham, 2018), and *tidyverse* (Wickham, 2017). All plots were created in *ggplot* (Wickham, 2009).

## 3 RESULTS

### 3.1 Parasitoid traits

**FIGURE 1:**
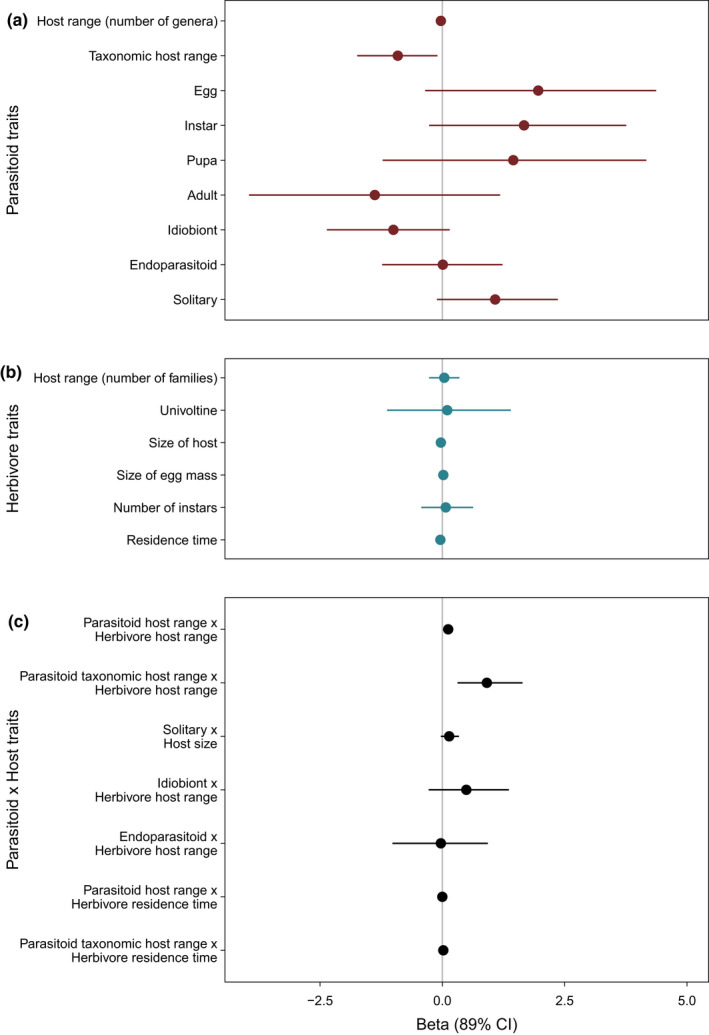
Forest plot depicting estimates of *β* (with 89% credible intervals) for (a) parasitoid traits, (b) herbivore traits, and (c) the interaction between parasitoid and herbivore traits. If the credible intervals overlap 0, the evidence that the trait or interaction of interest does not influence establishment success is high. If *β* is positive, a parasitoid is more likely to establish with that trait or with a greater value of that trait. If *β* is negative, as it is for phylogenetic host range (a), for example, a parasitoid is more likely to establish if it has a smaller phylogenetic host range

**FIGURE 2:**
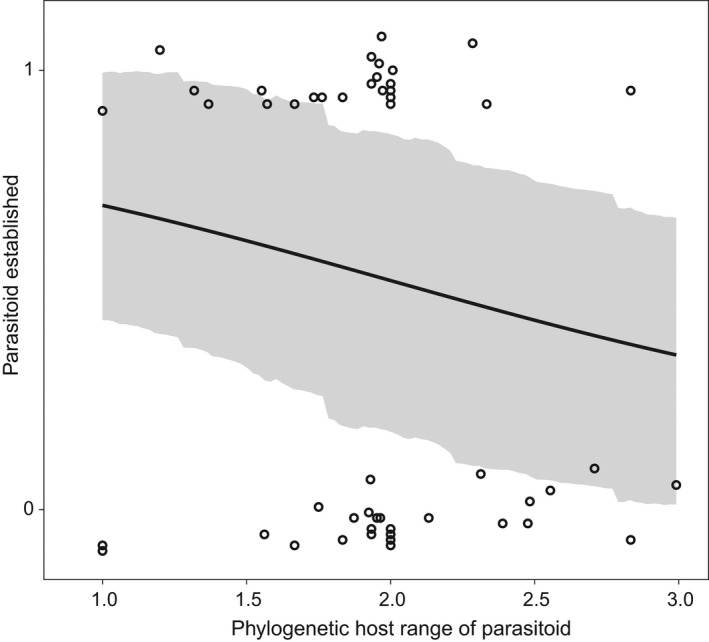
The probability of establishment for biological control agents decreases with the phylogenetic host range of the parasitoid. Solid black line indicates the predicted probability with 89% CIs shown in gray

We did not find overwhelming evidence that any one of the five parasitoid traits we analyzed on their own would predict variation in establishment success (Figure 1a). We found weak support that three parasitoid traits influenced the likelihood of parasitoid establishment. A smaller phylogenetic host range of the parasitoid species (i.e., a phylogenetic specialist) is associated with a greater likelihood of establishment (*β* = –0.91, [–1.74, –0.10], *N* = 95, Figures 1a and 2), though model comparison with the null model does not fully justify our confidence in the beta estimate; the model with phylogenetic host range is preferred with a lower WAIC (*Δ*WAIC = 0.19, SE = 3.97) score, though the standard errors are large; and the model weights are generally even split between the null model and the model containing the phylogenetic host range (WAIC weight of complex model = 0.52). Conversely, we found that parasitoid host range as measured by the number of genera they attack is not associated with the likelihood of establishment (*β* = –0.03, [–0.10, 0.05], *N* = 98, Figure 1a).

Second, we found some evidence that idiobiont parasitoids are less likely to establish (*β* = –1.00, [–2.36, 0.15], *N* = 91), but this is not consistently borne out when comparing the full model with the simplified model (ΔWAIC = 1.13, SE = 2.61), nor with the model weights (WAIC weight of complex model = 0.64). The third parasitoid trait that is suggested to increase establishment success of a parasitoid species is if the parasitoid is solitary (*β* = 1.08, [–0.11, 2.36], *N* = 97). The simplified model, however, has a lower WAIC score (ΔWAIC = 0.42, SE = 3.12).

We find that the host stage attacked by the parasitoid does not influence establishment success as all estimates overlap with 0 (egg: *β* = 1.96, [–0.35, 4.37]; instar: *β* = 1.67, [–0.27, 3.76]; pupa: *β* = 1.45, [–1.22, 4.17]; and adult: *β* = –1.38, [–3.95, 1.18], *N* = 96). The last trait we measured, whether the parasitoid was an endoparasitoid or ectoparasitoid, also did not predict variation in establishment success (*β* = 0.01, [–1.23, 1.23], *N* = 104).

### 3.2 Herbivore traits

We also found that, independently, none of the herbivore life‐history traits explain variation in establishment success of their parasitoids (Figure 1b). Whether the herbivore pest was a generalist or a specialist did not influence the establishment rate of the parasitoid released to control it (*β* = 0.04, [–0.27, 0.35], *N* = 111). The voltinism of the herbivore also did not affect establishment rate (*β* = 0.10, [–1.13, 1.40], *N* = 104). The size of the host that a parasitoid attacks also do not influence establishment rates (*β* = –0.03, [–0.09, 0.03], *N* = 67) and nor do the minimum number of instars a host goes through to complete development (*β* = 0.07, [–0.43, 0.63], *N* = 71).

**FIGURE 3:**
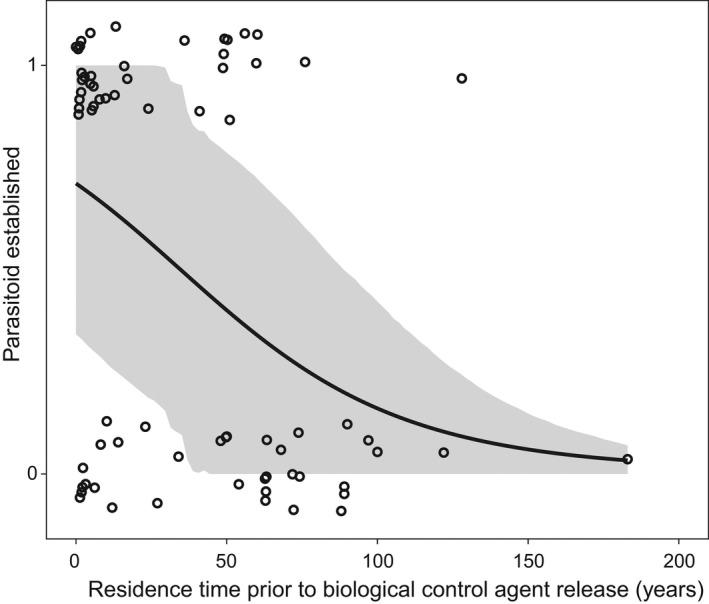
The probability of establishment for biological control agents decreases with the length of time between the first record of the invasive herbivore pest and the release of the agent. Solid black line indicates the predicted probability with 89% CIs shown in gray

There is some evidence that the minimum number of eggs in a host egg mass influences establishment success for egg and instar parasitoids (*β* = 0.02, [0.00, 0.04], *N* = 58), with larger egg mass sizes resulting in greater likelihood of establishment. Model comparisons and model weights, however, do not support this, as the simplified model has a lower WAIC value (ΔWAIC = 0.88, SE = 1.56), as well as no support from the model weightings (WAIC weight of complex model = 0.39). We find support that the residence time of the herbivore species is negatively correlated with the likelihood that a parasitoid species establishes. Chances of establishment are higher against hosts residing for shorter periods in the introduced range (*β* = –0.04, [–0.06, –0.02], *N* = 89, Figure 3), which are confirmed by model comparisons (ΔWAIC = 7.24, SE = 7.17) and model weighting (WAIC weight of complex model = 0.97).

### 3.3 Interactions between host and parasitoid traits

**FIGURE 4:**
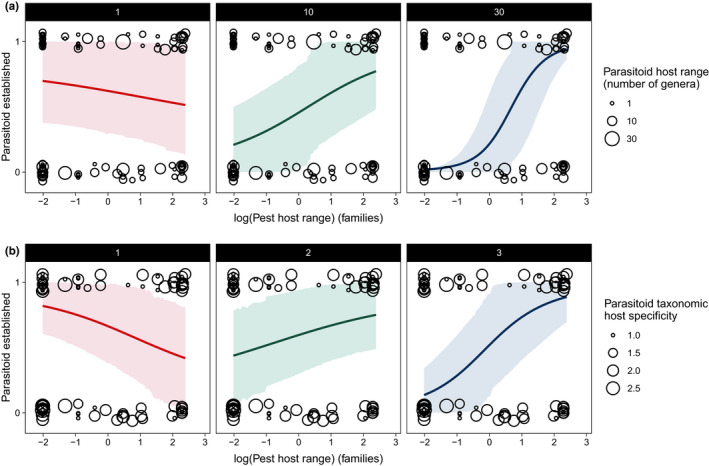
The relationship between the numerical host range (number of genera attacked by a parasitoid) of parasitoids and the host range of their target hosts for predicting establishment success of parasitoids in importation biological control programs (a). An alternative metric of parasitoid host range, parasitoid taxonomic host specificity, and its relationship with herbivore host range is also shown (b). Each circle represents a parasitoid species released that either established (1) or did not establish (0). The size of the circles represents the host range of the parasitoid species as a categorical variable of 1, 10, or 30 host genera attacked in A, or ranging from 1 to 2.5 in terms of taxonomic host specificity in B. All the raw data are displayed on each panel. The graphs show model outputs based on *N* = 100 fitted draws from the dataset for 3 hypothetical parasitoid species that attack 1, 10, or 30 host genera (a) or parasitoid species with a taxonomic host specificity of 1, 2, or 3 (b) with the lines indicating the likelihood of their establishment in relation to the host range of herbivores attacked. In the first panel of both a and b (red line), the model predicts that a specialist parasitoid that attacks only 1 host genus or taxonomic host specificity of 1 has a relatively high establishment probability independent of the host range of their host. In the second panel (green line), a parasitoid that attacks 10 genera (a) or has a taxonomic host specificity of 2 (b) has a higher probability of establishing when their host is a generalist. In the third panel (blue line), a generalist parasitoid that can attack 30 host genera (a) or has a taxonomic host specificity of 3 (b) has low probability of establishing on a specialist herbivore, but a high chance of establishing on a generalist herbivore. Shaded areas around the lines represent 89% credible intervals

Only one of our six a priori hypotheses regarding specific interactions between parasitoid and herbivore traits was supported. We found evidence to suggest that the (phylogenetic) host range of the parasitoid interacts with the host range of the herbivore species to determine whether a parasitoid species establishes (Figure 1c). Establishment rates of more specialized parasitoids (species that attack fewer herbivore genera) are similar on generalist and specialist hosts, but as the host range of parasitoids increases from specialists to generalists, the probability of establishing decreases except when their herbivore hosts are generalists as well (Figure 4a). The number of genera a parasitoid can parasitize and the number of families a herbivore can feed on both, interactively, influence whether a parasitoid is likely to establish or not (*β* = 0.12, [0.05, 0.21], *N* = 95, Figure 4a). The model with the interaction was preferred over the model without the interaction (ΔWAIC = 8.54, SE = 6.79) and held greater weight (WAIC weight of complex model = 0.99). This pattern also held for phylogenetic host range, which is another metric of parasitoid host range (*β* = 0.94, [0.31, 1.64], *N* = 92, Figure 4b). The model containing the interaction of parasitoid phylogenetic host range and herbivore host range is preferred over the model without the interaction (ΔWAIC = 6.68, SE = 6.28) and is preferred with respect to the model weights associated with both models (WAIC weight of complex model = 0.96).

Five of the hypothesized interactions between parasitoid and herbivore traits did not appear to be good predictors of parasitoid establishment success (Figure 1c). Specifically, we found no evidence (1) that the voltinism of the herbivore species interacts with the host range of the parasitoid (*β* = 0.00 [–0.20, 0.21], *N* = 91) or the phylogenetic host range of the parasitoid (*β* = –0.73 [–2.87, 1.39], *N* = 88) to explain variation of parasitoid establishment; (2) that establishment of solitary parasitoids would be more likely on small hosts (*β* = 0.14, [–0.03, 0.34], *N* = 43); (3) that idiobionts are more likely to establish on specialized herbivore hosts (*β* = 0.49, [–0.28, 1.36], *N* = 88); (4) that endoparasitoids are more likely to establish on specialists hosts (*β* = –0.03, [–1.02, 0.93], *N* = 101); and (5) that neither the parasitoid host range (*β* = 0.00, [0.00, 0.01], *N* = 79) nor phylogenetic host range (*β* = 0.02, [–0.03, 0.08], *N* = 77) interact with the residence time of the herbivore host to explain variation in establishment success.

## 4 DISCUSSION

Here, we show that not only can individual parasitoid or herbivore traits explain variation in establishment success, but also certain species’ traits across trophic levels can interact to influence the likelihood of successful parasitoid establishment. In addition, we find that the residence time of hosts in the introduced range is an important predictor whether imported parasitoids establish. These results provide insights into the mechanisms that mediate community assembly in a rapidly changing world where alien species’ introductions, both unintentional and intentional, have become increasingly common.

### 4.1 Parasitoid traits

We found weak support that specialist parasitoids are more likely to establish than generalist parasitoids (Figures 1a and 2), a pattern identified by two previous studies (Kimberling, 2004; Rossinelli & Bacher, 2014). Rossinelli and Bacher (2014) used a dataset that included only parasitoid traits without host traits or their interaction and found that besides release size, dietary specialization explained best establishment of parasitoids released for biocontrol. An earlier study that used a >30‐year‐old dataset showed that a higher percentage of specialist (67%) than generalist (59%) parasitoids established, though the difference was not statistically significant (Stiling, 1990). Kimberling (2004), who looked at success of the parasitoids at controlling targets, a measure which can be highly correlated with establishment, also found that specialist parasitoids were more likely to be successful than generalists.

Several mechanisms may be evoked to explain the above findings. Specialists can be more efficient at locating their hosts and attacking them than generalist parasitoids because of their long‐term history of coevolution. Rossinelli and Bacher (2014) suggested that the higher establishment rates of specialists are due to fitness trade‐offs that arise with diet breadth; a smaller diet breadth could allow other traits to be better optimized for a specific host. Generalist parasitoids, however, could be argued to be more likely to establish (Symondson et al., 2002). First, generalists may be more able to cope with novel environments as they ostensibly occupy a large ecological niche. Second, generalists can utilize alternative hosts when there is a scarcity of target hosts, which could prevent extinction and help maintain viable population sizes. According to our analyses, the effect of parasitoid host range is better explained when considering the herbivore host range in concert, which can also resolve the seemingly contradictory explanations above (see section on interactions).

For all other individual parasitoid traits our analyses showed no explanatory power or only weak support in explaining establishment success. For example, we did not find evidence that the fecundity of the herbivorous host and whether the parasitoid was an endoparasitoid or ectoparasitoid would explain variation in establishment rate, as opposed to Stiling (1990). In addition, we found no support that gregarious parasitoids are more likely to establish than solitary parasitoids (Mills, 2001). If anything, our results tentatively suggest the opposite; that solitary parasitoids are more likely to establish (Figure 1a), despite the fact that in theory, the population growth of gregarious parasitoids could be faster and aid establishment (Mills, 2001). There was some indication that koinobionts may be more likely to establish (Figure 1a), which is consistent with the idea that koinobionts are more specialized (Godfray, 1994; Quicke, 1997), though that is not the case in our dataset (Figure S1). Whether a parasitoid is an idiobiont or koinobiont does not interact with herbivore host range the same way as parasitoid host range and phylogenetic host range does (Figure 4), which suggests the idiobiont/koinobiont dichotomy does not capture the same variation as a more direct measure of host range.

Some of the contradictory findings are likely due to the different datasets used in the above studies that varied in geographic scope, date range, and in the types of analyses undertaken, which failed to account for variation in random effects like phylogeny and year of release. Our database only covers North America, but it contains the most recent information (1985–2018) largely excluding years when documentation of biocontrol releases was less accurate. Thus, it is likely that our results reflect patterns that are valid for the Nearctic region. Nevertheless, it would be valuable to use worldwide databases such as BIOCAT (Cock et al., 2016) to compare assembly patterns across continents with the inclusion of environmental variables, such as climate, which can influence parasitoid establishment (Fischbein et al., 2019), as well as variables already known to influence establishment, like propagule size (Lockwood et al., 2005).

### 4.2 Host traits

Our results do not indicate that a wide range of host traits by themselves would predict parasitoid establishment, which is in contrast to Stiling (1990) who showed that host fecundity, voltinism, mobility, and habitat can all be important. While not a biological trait, we found evidence that the residence time of the invasive herbivorous pest influenced the establishment of the parasitoid released to control it (Figure 3): parasitoids released soon after the pest was discovered were more likely to establish. There is evidence that exotic herbivores in a new environment start accumulating native natural enemies and that the richness of the acquired enemy complex increases over time (Cornell & Hawkins, 1993). Competition with an increasing number of native natural enemies or increasing intraguild predation over time are two factors that might explain the negative relationship between parasitoid establishment and host residence time. Host evolution postinvasion could also explain this result, as longer residence times would be more likely to result in local adaptation (Dietz & Edwards, 2006, but see Oduor et al., 2016). Given that the acquired native natural enemy complexes are made up mostly of generalists (Cornell & Hawkins, 1993), we expected that establishment of specialist parasitoids would be less affected by the host's residence time than that of generalists that may more directly compete with native species, but this was not the case (Figure 1c).

### 4.3 Interaction of parasitoid and host traits

We found that generalist parasitoids are more likely to establish when their target is a generalist as well, but specialist parasitoids are equally likely to establish on either a specialist or a generalist host. Generalist parasitoids attack a wide range of different species that most likely occupy different niches, including feeding on a range of host plants. The search behavior of generalist parasitoids involves moving from patch to patch more rapidly than specialist parasitoids (Kimberling, 2004). When host species are also generalists and are spread across patches of many different plant species, this behavior could aid establishment, with some evidence from life tables to suggest generalists do provide greater top‐down control in native habitats with a greater range of host plants (Hawkins et al., 1999). On the other hand, specialist hosts would only be found in a particular patch type, which could be harder to detect with the random search behavior of generalist parasitoids. This simplified network of one host plant for specialist herbivores, especially in a cultivated landscape, is more likely to involve top‐down control from specialist parasitoids (Hawkins et al., 1999). There are also differences in parasitism rate for generalist and specialist herbivores, where parasitism is higher for specialists than it is for generalists (Dyer & Gentry, 1999; Gentry & Dyer, 2002), which could mean that competition for generalist hosts is lower and thus presents less competition for generalist parasitoids released to control them.

Another, nonmutually exclusive explanation is that specialist parasitoids have coevolved with plant species (including imported crops) such that when a particular plant species is attacked by a herbivore, it releases herbivore‐induced plant volatiles (Abdala‐Roberts et al., 2019; Price et al., 1980; Turlings & Erb, 2018; Vet & Dicke, 1992). In many cases, the released volatiles attract parasitoid species that are specialists on the specific species attacking the plant (Blande et al., 2007; De Moraes et al., 1998; McCormick et al., 2012). These tritrophic interactions between plants and their herbivore's natural enemies are one potential mechanism that explains why specialist parasitoids are able to establish if their hosts are either generalists or specialists. Generalist parasitoids, however, generally do not discriminate between specific herbivore‐induced plant volatiles and would therefore dampen the coevolutionary dynamics between the three trophic levels, potentially leading to a lower probability of establishment (McCormick et al., 2012).

### 4.4 Limitations of the study

For our analysis exploring how traits across trophic levels might interact in affecting the outcomes of biological control agent establishment, we used a recent, publicly available dataset (Van Driesche et al., 2018). While an excellent resource, Van Driesche et al. (2018) is limited to North American biological control releases in the last 35 years and thus covers only a small subset of the biological control releases that have occurred throughout history. The total sample size for this dataset is *N* = 132, but by including various traits, sample sizes for each analysis fluctuated between 43 and 111. Any relationships that we tested may be biologically real but weak were underpowered. Testing ideas using a larger database of biological control releases, like BIOCAT (Cock et al., 2016), would provide greater power and more definite conclusions about the role of cross‐trophic trait interactions in biological control agent establishment success. In addition, we used Van Driesche et al. (2018) for our estimates of parasitoid host range. Van Driesche et al. (2018) collated such data from the literature and from information gathered from specialists within the field, but using further estimates of parasitoid host range from alternative sources would result in a more robust dataset free from any potential bias a single source of any information might include, like differences in taxonomic classification.

Current methods of reporting establishment success and subsequent control should also include and report the uncertainty in any estimate of establishment or control. Distilling all the relevant information into a single binary outcome (“established” vs “not establishment”, or “control achieved” vs “no control achieved”), while easy to analyze, is a form of vote‐counting (Haddaway et al., 2020). When assessing establishment or control of a biological control agent, including the sampling effort would allow more powerful, meta‐analytic methods to be employed when analyzing such data.

Further limitations of our study that should be taken into account when examining our results include the relative paucity of data. For example, the host range of the herbivore pests could only be collected at the family level for the majority of species, producing a disconnect between the host range information of the herbivores and the host range data of their parasitoids. Such data are hard to accumulate, especially across a wide range of taxa that spans four insect orders. Missing data, too, contribute to the changes in sample sizes across analyses, and if such missing data are biased taxonomically (i.e., certain subfamilies are less well studied), biased conclusions may be reached.

### 4.5 Implications for biological control

Biological control releases can shed light onto fundamental biological processes, like community assembly and invasion, even if they represent a special case (Abram & Moffat, 2018; Hawkins et al., 1999; Holt & Hochberg, 2001; Yeates et al., 2012). Our work adds to this growing literature by being the first to explicitly investigate the interactive effects of life‐history variation between two trophic levels on establishment success of biological control agents.

Importation biological control releases are, however, primarily undertaken to control pest species for economic reasons. As such, selection of biological control agents is not random with respect to life‐history trait variation, since the aim is to select a coevolved parasitoid with the greatest chance of success. Yet, there is a great variation in biological control release success despite such meticulous planning, and analyses like ours seek to understand the mechanisms underlying this variation in the hope of uncovering specific traits, or trait combinations, that will further improve biological control success in future. Our results indicate that a specialist is more likely to establish in biological control releases especially if the herbivore is also a specialist, but when it comes to a generalist pest, a generalist parasitoid has a greater chance of establishing (Figure 2). While releasing a generalist parasitoid to combat a generalist pest may be advantageous in promoting establishment, it also increases the risk of nontarget effects. The majority of introduced natural enemies in Hawai'i that have successfully established are generalists that dominate food webs and attack a range of nontarget species as well as the target herbivorous pest they were released to control (Henneman & Memmott, 2001; Kaufman & Wright, 2009).

While in the past the release of generalist parasitoids may have been permitted, current regulations would allow in most cases only highly specialized parasitoid species to be released (Heimpel & Cock, 2018; Hoddle, 2004). Thus, planned releases of generalist parasitoids for importation biological control are unlikely to happen in future. Nevertheless, natural enemies occasionally follow invasive species and adventive populations of biological control agents that can be generalists may show up in the exotic range (e.g Beltra et al., 2013; Heimpel et al., 2010; Stahl et al., 2019). In these instances, the primary question for the practice of biological control is whether to promote the establishment and spread of these natural enemies across the geographic range of the pest to speed up control. A recent example for such a conundrum is the invasion of a generalist pest, the brown marmorated stink bug (*Halyomorpha halys*) into North America and Europe. This was followed by the invasion of one of its closely associated parasitoid, *Trissolcus japonicus*, which can attack multiple genera of stink bugs including many native species in the introduced range (Botch & Delfosse, 2018; Hedstrom et al., 2017; Milnes & Beers, 2019; Stahl et al., 2019). Our results indicate that for a generalist pest such as *H. halys*, an oligophagous parasitoid such as *T. japonicus* has a relatively good chance of establishing and potentially offer some level of biological control. However, this control may come at the expense of nontarget effects, and thus, the risks and benefits will need to be balanced in situations like this (Louda et al., 2003). Inclusion of adventive populations of exotic parasitoids into analyses such as ours would provide an interesting contrast to those preselected by biological control practitioners.

Finally, even biological control agents that are relatively new members of most communities will start accumulating natural enemies. In New Zealand, a suite of native parasitoids was found to attack exotic herbivorous insects released to control invasive weeds (Paynter et al., 2010). The population‐level impact of these recently acquired natural enemies were large enough to reduce the effectiveness of the herbivores at providing weed biocontrol (Paynter et al., 2010). Parasitoids released against insect pests are likely to accumulate natural enemies themselves (e.g., Broadley et al., 2018), including hyperparasitoids (Hofsvang et al., 2014), and as discussed previously, they are likely to compete with native parasitoids that have started adopting the exotic pests as hosts. The complex food webs and intricate biotic interactions that develop around introduced biocontrol agents will influence the effectiveness of biocontrol and can serve to provide unique insights how multitrophic interactions shape invasion success.

However, this complexity might also mean that search for any trait‐based patterns to predict control or establishment success of biocontrol agents may be elusive. The contradictory findings of different studies, the large variability in the data, and the weak or lack of statistical support for most hypotheses mean that reliable predictions may not exist for applied biological control. Using larger datasets may not remedy this problem if the reality is that a trait‐based approach may just simply not work for classical biological control.

## CONFLICT OF INTEREST

The authors declare no conflict of interest.

## AUTHOR CONTRIBUTIONS

**Benjamin J. M. Jarrett:** Conceptualization (lead); Data curation (lead); Formal analysis (lead); Investigation (lead); Methodology (lead); Writing – original draft (lead); Writing – review & editing (equal). **Marianna Szucs:** Conceptualization (supporting); Funding acquisition (lead); Supervision (lead); Writing – original draft (supporting); Writing – review & editing (equal).

## Supporting information

Appendix S1

Fig S1

Fig S2

Fig S3

Fig S4

## Data Availability

All data and exemplar code are available on Dryad (https://doi.org/10.5061/dryad.80gb5mksj).
