# Supplementary material for: Traits across trophic levels interact to influence parasitoid establishment in biological control releases
Source: Ecol Evol. 2022 Mar 8;12(3):e8654. doi: 10.1002/ece3.8654 (PMC8928891; doi:10.1002/ece3.8654)
Supplement: Supplementary file 1 — Appendix S1 [file ECE3-12-e8654-s001.docx]

**Appendix S1**

**Parasitoid and host cladograms**

Parasitoid cladogram construction

Cladograms for both the parasitoid species and herbivore species were created by first searching Time- Tree.org for a framework. For the parasitoids, this provided a tree of the Hymenopteran families from which our species originated. The Dipteran families were then added. We focused on the branching patterns of our focal species as branch length data for our list of taxa is not available. We therefore set the branch lengths using the Grafen method in the R package *ape* (Paradis, Claude, and Strimmer 2004) such that the tree is ultrametric. Randomised branch lengths did not influence the beta coefficients and credible intervals of our models (Supplementary Figure 3). Using the skeleton from TimeTree.org, we focused on specific families and subfamilies to provide more detail. We assumed all genera (unless found otherwise) were monophyletic and so placed species in with the genus, even if our focal species was not used to construct the phylogeny. In many cases we could not find published phylogenies within genera; in these cases, we used the genus as a polytomy where all species are assumed to be equally related to each other. We also used this method for monophyletic families (Bethylidae, Pteromalidae and Encyrtidae within the Hymenoptera, and Tachinidae and Phoridae in the Diptera) and one sub-family (Opiinae within the Braconidae). We used Munro et al. (2011) for the majority of the Chalcidoidea, with further information on the Eulophidae from Gauthier et al. (2000). The Ichneumonidae were assessed using Quicke et al. (2009), and Murphy et al. (2007) for the Scelionidae and Platygastridae. To ascertain the relationships between the subfamilies of the Braconidae, we used Dowton, Austin, and Antolin (1998), with more detailed phylogenies for the Aphidiinae (Belshaw and Quicke 1997; Sanchis et al. 2000), Doryctinae (Zaldivar-Riverón et al. 2008), and Euphorinae sub-families (Stigenberg, Boring, and Ronquist 2015).

Host cladogram construction

We followed the same protocol as with the parasitoid cladogram by using TimeTree.org as a base. We used Hunt et al. (2007) to determine the relationships between Coleopteran families. Information from TimeTree.org was lacking with respect to the Hemiptera. We therefore used Li et al. (2017) as a guide for relationships between families, and consulted Cho, Malenovsky, and Lee (2019) for the Psylloidea, Gullan and Cook (2007) for the Sternorrhyncha, Thao and Baumann (2004) for the Aleyroididae, Morse and Normark (2006) for the Diaspididae, and Downie and Gullan (2004) for the Psuedococcidae.

**References**

Belshaw R, Quicke DL (1997) A molecular phylogeny of the Aphidiinae (Hymenoptera: Braconidae). *Molecular Phylogenetics and Evolution* 7: 281–293.

Cho G, Malenovsky I, Lee S (2019) Higher-level molecular phylogeny of jumping plant lice (Hemiptera: Sternorrhyncha: Psylloidea). *Systematic Entomology* 44: 638–651.

Downie D, Gullan P (2004) Phylogenetic analysis of mealybugs (Hemiptera: Coccoidea: Pseudococcidae) based on DNA sequences from three nuclear genes, and a review of the higher classification. *Systematic Entomology* 29: 238–260.

Dowton M, Austin A, Antolin A (1998) Evolutionary relationships among the Braconidae (Hymenoptera: Ichneumonoidea) inferred from partial 16S rDNA gene sequences. *Insect Molecular Biology* 7: 129–150.

Gauthier N, et al. (2000) Phylogeny of Eulophidae (Hymenoptera: Chalcidoidea), with a reclassification of Eulophinae and the recognition that Elasmidae are derived eulophids. *Systematic Entomology* 25: 521–539.

Gullan P, Cook L (2007) Phylogeny and higher classification of the scale insects (Hemiptera: Sternorrhyncha: Coccoidea). *Zootaxa* 1668: 413–425.

Hunt T, et al. (2007) A comprehensive phylogeny of beetles reveals the evolutionary origins of a superradiation. *Science* 318: 1913–1916.

Li H, et al. (2017) Mitochondrial phylogenomics of Hemiptera reveals adaptive innovations driving the diversification of true bugs. *Proceedings of the Royal Society B: Biological Sciences* 284: 20171223.

Morse GE, Normark BB (2006) A molecular phylogenetic study of armoured scale insects (Hemiptera: Diaspididae). *Systematic Entomology* 31: 338–349.

Munro JB, et al. (2011) A molecular phylogeny of the Chalcidoidea (Hymenoptera). *Plos one* 6 (11).

Murphy NP, et al. (2007) Phylogeny of the platygastroid wasps (Hymenoptera) based on sequences from the 18S rRNA, 28S rRNA and cytochrome oxidase I genes: implications for the evolution of the ovipositor system and host relationships. *Biological Journal of the Linnean Society* 91: 653–669.

Paradis E, Claude J, Strimmer K. (2004) APE: analyses of phylogenetics and evolution in R language. *Bioinformatics* 20 (2): 289–290.

Quicke DL, et al. (2009) A thousand and one wasps: a 28S rDNA and morphological phylogeny of the Ichneumonidae (Insecta: Hymenoptera) with an investigation into alignment parameter space and elision. *Journal of Natural History* 43: 1305–1421.

Sanchis A, et al. (2000) An 18S rDNA-based molecular phylogeny of Aphidiinae (Hymenoptera: Braconidae). *Molecular Phylogenetics and Evolution* 14: 180–194.

Stigenberg J, Boring CA, Ronquist F (2015) Phylogeny of the parasitic wasp subfamily Euphorinae (Braconidae) and evolution of its host preferences. *Systematic Entomology* 40: 570–591.

Thao ML, Baumann P (2004) Evidence for multiple acquisition of *Arsenophonus* by whitefly species (Sternorrhyncha: Aleyrodidae). *Current Microbiology* 48: 140–144.

Zaldivar-Riverón A, et al. (2008) Molecular phylogeny and historical biogeography of the cosmopolitan parasitic wasp subfamily Doryctinae (Hymenoptera: Braconidae). *Invertebrate Systematics* 22: 345– 363.
