# Supplementary figures and images for: Traits across trophic levels interact to influence parasitoid establishment in biological control releases
Source: Ecol Evol. 2022 Mar 8;12(3):e8654. doi: 10.1002/ece3.8654 (PMC8928891; doi:10.1002/ece3.8654)

A

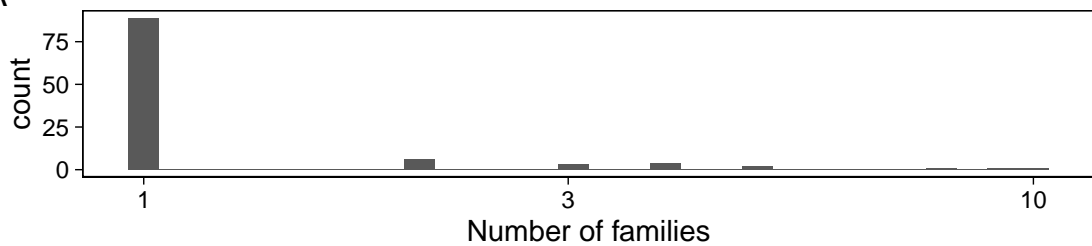

B

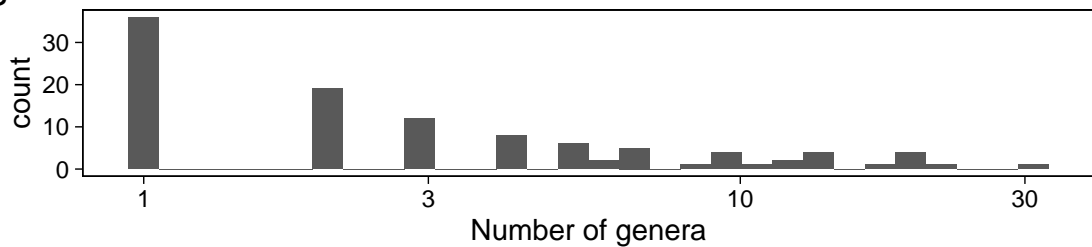

C

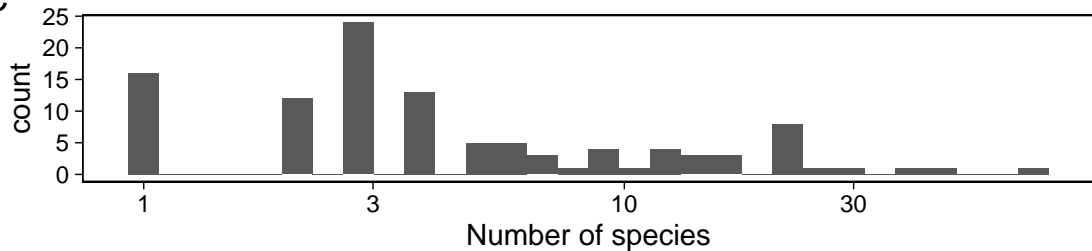

D

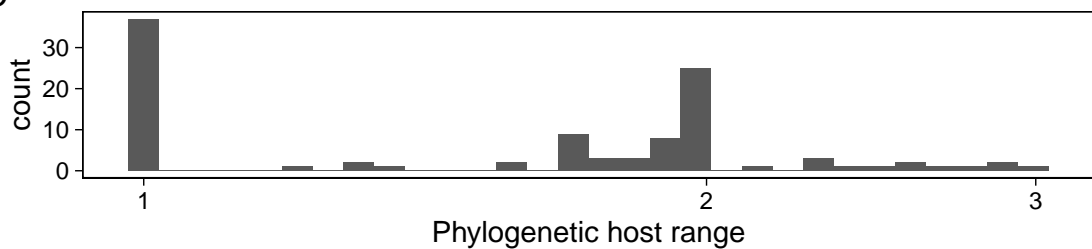

Supplement: Supplementary file 2 — Fig S1 [file ECE3-12-e8654-s004.pdf]

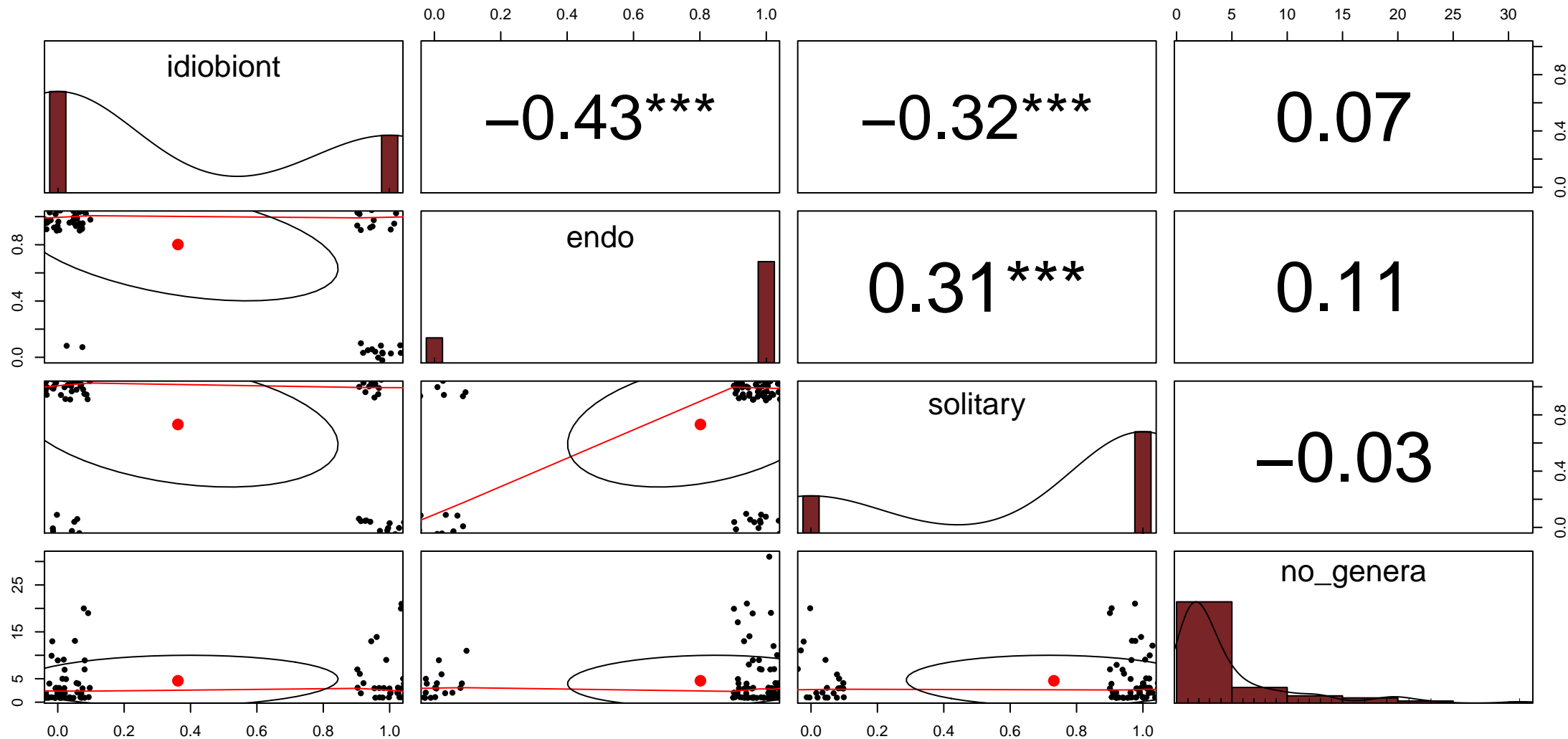

Supplement: Supplementary file 3 — Fig S2 [file ECE3-12-e8654-s002.pdf]

Parasitoid tree

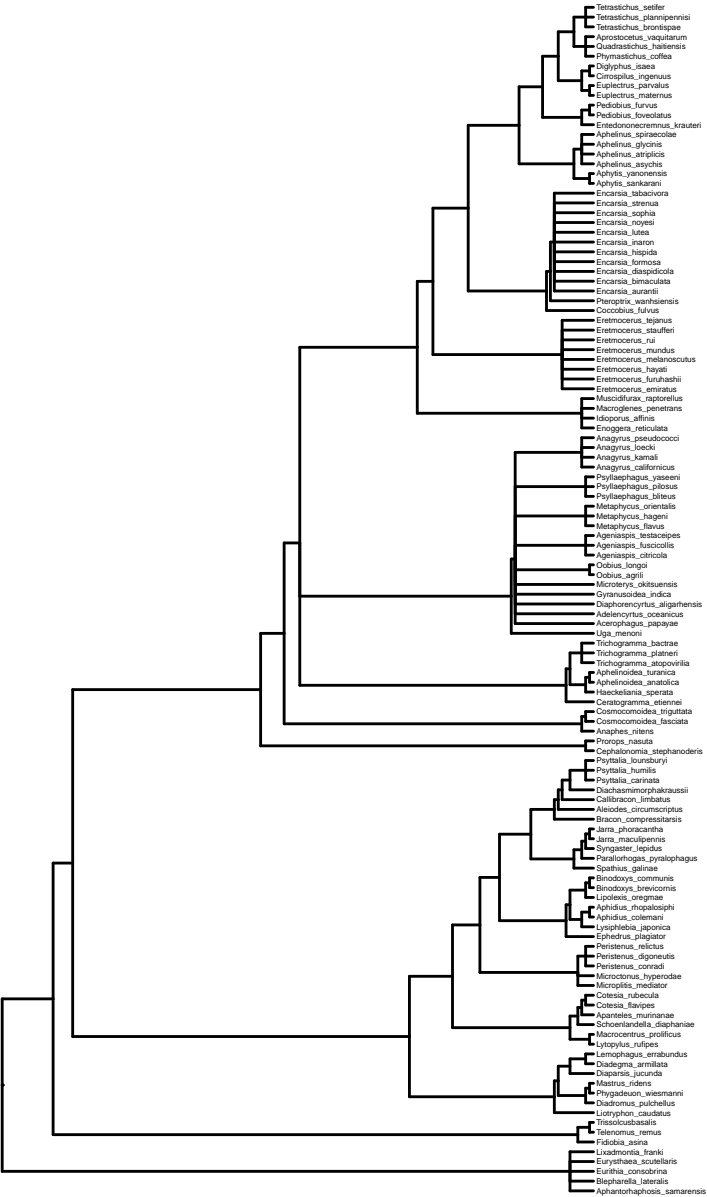

Herbivore tree

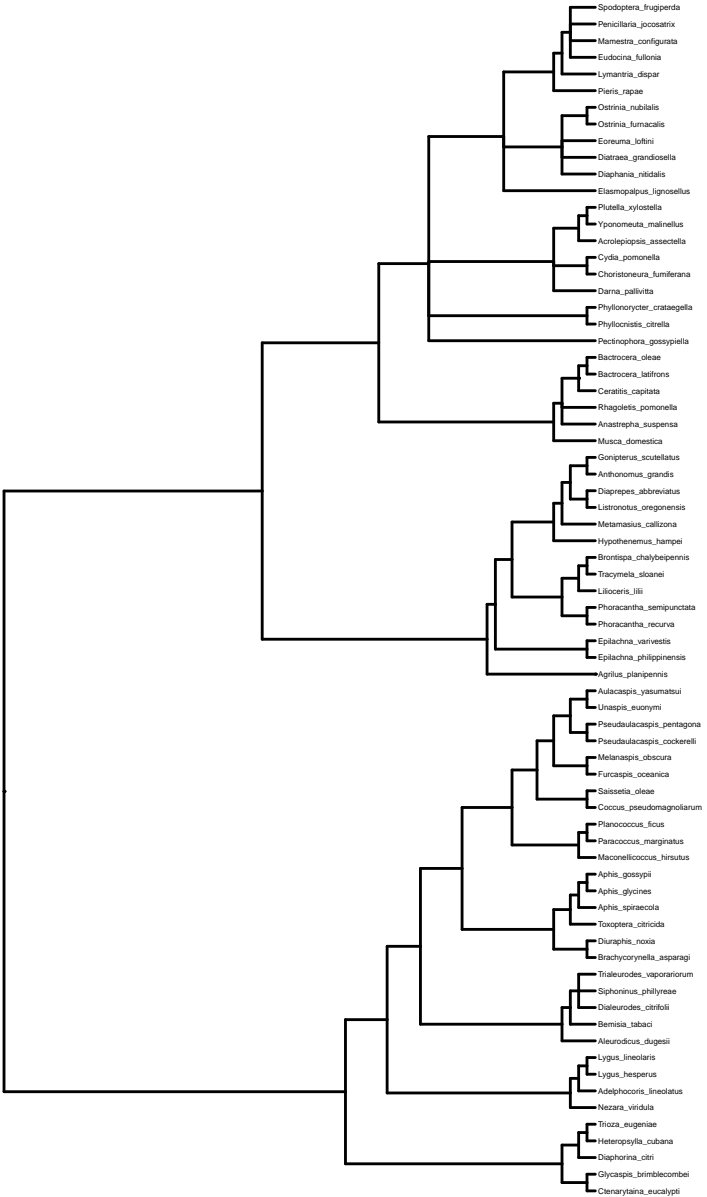

Supplement: Supplementary file 4 — Fig S3 [file ECE3-12-e8654-s005.pdf]

A

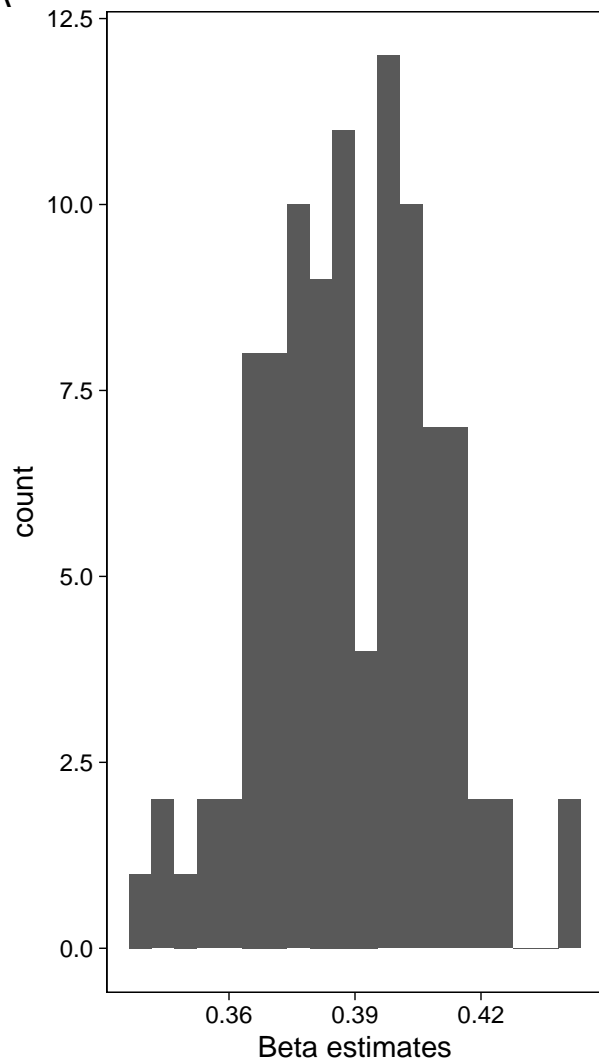

B

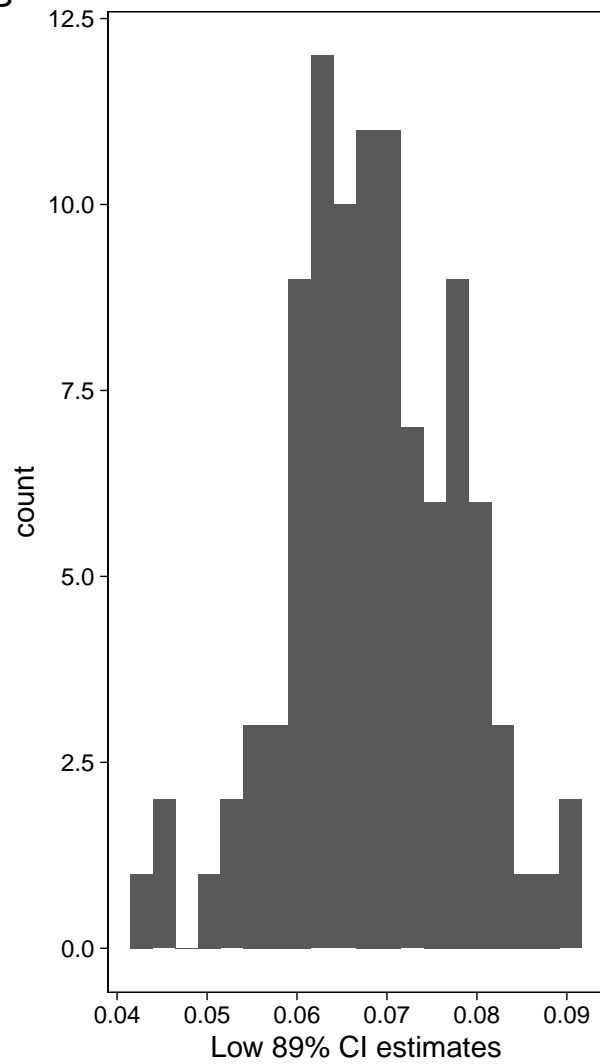

C

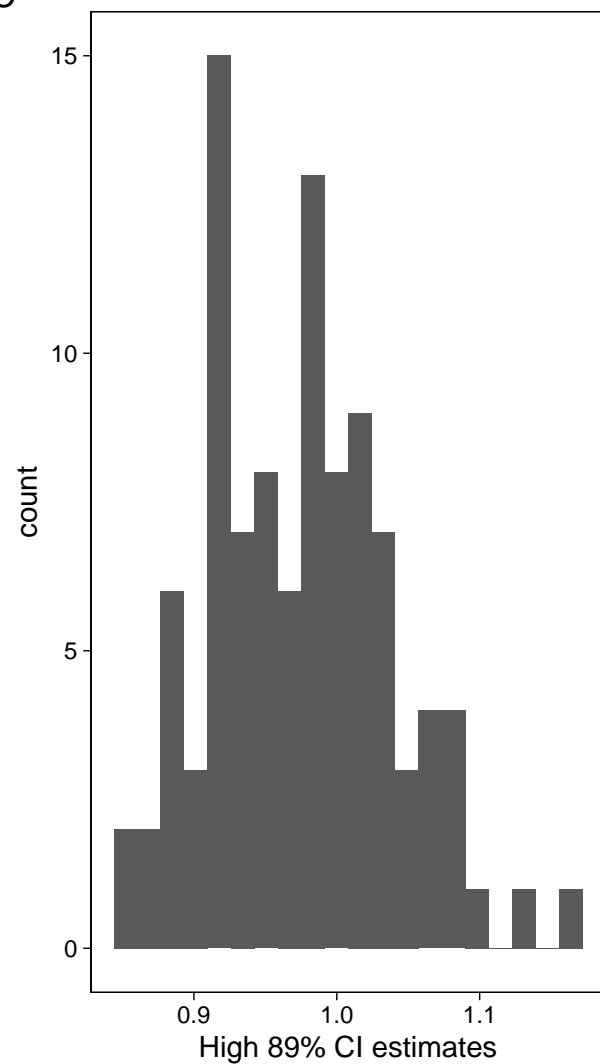

Supplement: Supplementary file 5 — Fig S4 [file ECE3-12-e8654-s003.pdf]
